# Beyond COVID-19: the promise of next-generation coronavirus vaccines

**DOI:** 10.1038/s44298-024-00043-3

**Published:** 2024-08-22

**Authors:** Reshma Koolaparambil Mukesh, Claude K. Yinda, Vincent J. Munster, Neeltje van Doremalen

**Affiliations:** Laboratory of Virology, Division of Intramural Research, National Institutes of Health, Hamilton, MT USA

**Keywords:** SARS-CoV-2, Vaccines

## Abstract

Coronaviruses (CoVs) have caused three global outbreaks: severe acute respiratory syndrome coronavirus 1 (SARS-CoV-1) in 2003, Middle East respiratory syndrome coronavirus (MERS-CoV) in 2012, and SARS-CoV-2 in 2019, with significant mortality and morbidity. The impact of coronavirus disease 2019 (COVID-19) raised serious concerns about the global preparedness for a pandemic. Furthermore, the changing antigenic landscape of SARS-CoV-2 led to new variants with increased transmissibility and immune evasion. Thus, the development of broad-spectrum vaccines against current and future emerging variants of CoVs will be an essential tool in pandemic preparedness. Distinct phylogenetic features within CoVs complicate and limit the process of generating a pan-CoV vaccine capable of targeting the entire *Coronaviridae* family. In this review, we aim to provide a detailed overview of the features of CoVs, their phylogeny, current vaccines against various CoVs, the efforts in developing broad-spectrum coronavirus vaccines, and the future.

## Introduction

Vaccines are safe and effective biological formulations synthesized to protect the population from infectious diseases by preventing, limiting, or eradicating the infection and spread of a specific pathogen by inducing specific immune responses^1^. The design and development of an effective vaccine against infectious diseases requires considerable knowledge about the causative agent, including the nature of the infection, phenotypic characteristics, and infection-associated pathogenesis. The safety, broad-spectrum efficacy, ease of administration, induction of long-term immunity, minimal cost of production, and extended shelf life through improved thermostability are other factors of paramount importance in developing an ideal vaccine^2^. The medical, social, and economic burden caused by COVID-19 and the evolution and emergence of new variants has highlighted the urgent need for next-generation vaccines with increased breadth of protection, durability, and ability to block infection and transmission.

CoVs belong to the *Coronaviridae* family and are known to cause outbreaks on endemic and pandemic scales. These viruses are genetically diverse and distinct as demonstrated by phylogenetic analysis and their evolutionary origin. To date, seven human CoVs (HCoVs) have been identified, namely HCoV-229E, HCoV-OC43, HCoV-NL63, HCoV-HKU1, SARS-CoV-1, MERS-CoV, and SARS-CoV-2^3–8^. Studies focused on the origin of these viruses have shown that bats, rodents, and domestic animals serve as the main reservoir species^9^. Concerns regarding the possibility of future spillovers with unknown CoVs and the emergence of SARS-CoV-2 variants of concern (VOCs) with unique mutations necessitate generating broad-spectrum vaccines. In addition, waning immunity and breakthrough infections within the population strongly advocate for developing improved vaccines with increased immunogenicity and durability. The first-generation vaccines designed to prevent disease with the ancestral SARS-CoV-2 were proven to be less effective against VOCs. For instance, multiple studies have shown that neutralizing antibody responses induced by the first-generation COVID-19 vaccines were limited against most of the Omicron variants^10–16^. Waning immunity against SARS-CoV-2 in the population necessitates the need for frequent boosters^17–19^.

Vaccine design and the delivery route are essential aspects to consider while developing a vaccine. SARS-CoV-2 vaccine design mainly focuses on two antigen targets: the spike (S) protein and the receptor binding domain (RBD) in the S protein. Both targets result in the induction of neutralizing antibody responses^20,21^. The S protein amino acid sequence similarity between SARS-CoV-1 and SARS-CoV-2 is 76%, and between SARS-CoV-2 and SARS-related CoVs (SARSr-CoVs) is ~80%^22^. Likewise, the RBDs of SARS-CoV-1 and ancestral SARS-CoV-2 have an amino acid sequence similarity of 73.5%^23^. Hence, in the design of pan-CoV vaccines, it is wise to consider S protein and RBDs from different CoVs.

Available COVID-19 vaccines are administered intramuscularly (IM) and provide robust systemic antibody responses. Since CoVs are respiratory pathogens and the primary route of virus entry is through the upper respiratory tract, inducing a local mucosal immune response could be an effective way to protect against CoV infection and transmission. Studies focusing on mucosal vaccines have shown induction of mucosal, cellular, and humoral immune responses^24^. Transmission still occurs from IM-vaccinated individuals^25–27^, and combining mucosal and IM vaccination routes could reduce or completely prevent virus transmission^28–30^.

### Coronaviruses—an overview

Coronaviruses are enveloped, positive-sense, single-stranded RNA viruses belonging to the order *Nidovirales* and the family *Coronaviridae*. The family *Coronaviridae* is further divided into three subfamilies: *Letovirinae, Pitovirinae, and Orthocoronavirinae*, the latter of which is the focus of this review. The subfamily *Orthocoronavirinae* is subdivided into four genera: Alpha, Beta, Gamma, and Deltacoronaviruses. SARS-CoV-1, SARS-CoV-2, and MERS-CoV belong to the Betacoronavirus genera^31^.

The general structure of the virus is spherical, with crown-like protrusions present on the outer surface of the virus, which are formed by the S protein, required for the attachment, fusion, and entry of the virus into the host cell. Besides S, CoVs are made of additional structural proteins: the membrane protein (M), the envelope protein (E), and the nucleocapsid protein (N). Both M and E proteins play major roles in viral assembly^32,33^. HCoV-OC43 and HCoV-HKU1 contain an additional structural protein called the hemagglutinin-esterase protein (HE)^34^. These proteins, together with the lipid bilayer, form the viral envelope seen surrounding the helical capsid formed by N. The large genome of CoVs, typically 22 to 36 kb, is packaged within the capsid^32,35^.

Coronaviruses can cause a myriad of diseases both in animal and human populations. Animal CoVs target various animals, including cows, pigs, dogs, cats, mice, and chickens, and can cause gastroenteritis, encephalitis, and bronchitis. For instance, murine hepatitis virus (MHV) causes respiratory, enteric, hepatic, and neurologic infections in mice^36,37^. Although there are multiple animal CoVs, we focus on HCoVs in this review. HCoV-229E, HCoV-NL63, HCoV-OC43, and HCoV-HKU1 cause mild respiratory tract infections mainly in immunocompromised individuals, which are self-limiting^4–6,38^. However, infection with SARS-CoV-1, MERS-CoV, and SARS-CoV-2 can affect multiple organs, and patients can develop acute respiratory distress syndrome (ARDS), extrapulmonary manifestations, and multiple organ dysfunction syndrome (MODS)^39^.

The presentation of COVID-19 can either be asymptomatic or symptomatic, ranging from mild to moderate to severe^40–45^. Lung damage is directly proportional to the virus replication and the titer, immune cell infiltration, and elevated levels of various proinflammatory cytokines and chemokines^46–50^. The immunological response, including the induction of a cytokine storm, can lead to severe lung damage and increased mortality^47,51^. Several COVID-19 survivors have prolonged health complications associated with COVID-19, often referred to as long COVID^52^.

### The phylogeny and evolution of coronaviruses

Acquisition of mutations and recombination within the virus genome are major driving forces that create genetic diversity, eventually aiding in increased virus survival, pathogenicity, transmissibility, and immune evasion^53^. CoVs have an internal proofreading mechanism: the 3′ exonuclease (nsp14 for SARS-CoV-2), which limits the mutation rate of CoVs^54,55^. It has been estimated that the mutation rate of CoVs is 10^−6^ per base per infection cycle^56^ and 10^-3^ per site per year^57^. Recombination events in conjunction with purifying selection result in the generation of new CoV variants^58,59^.

**Fig. 1:**
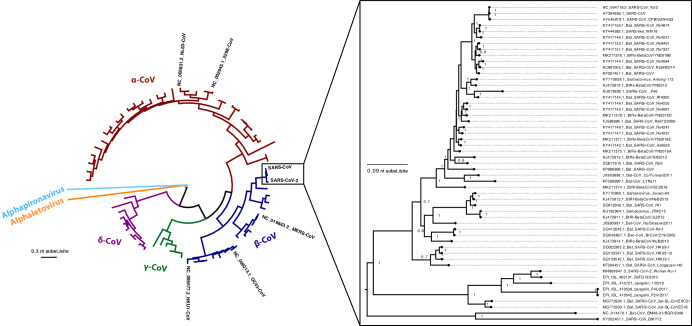
Maximum likelihood phylogenetic trees showing sequences of representative members of the family *Coronaviridae* (circular tree) and all representative members of the Sarbecovirus subgenera (rectangular tree). Full-length genomes were obtained from GenBank (https://www.ncbi.nlm.nih.gov/genbank/), and alignments were built with MAFFT (FFT-NS-1 algorithm^181^. Maximum likelihood phylogenetic tree was constructed using IG-TREE2^182^ with the best model for distance estimates identified with the ModelFinder function^183^ as the one with the lowest Bayesian information criterion (BIC). Branch support was assessed using both ultrafast bootstrap approximation (ufBoot, 1000 replicates)^184^ and SH-like approximate likelihood ratio test (SH-aLRT). The tree was visualized in FigTree (http://tree.bio.ed.ac.uk/software/figtree/), and midpoint rooted for purposes of clarity. Only bootstrap values greater than 70% are shown. Bars indicate nucleotide substitutions per site.

The comparative genomic analysis of MERS-CoV, SARS-CoV-1, and SARS-CoV-2 shows that SARS-CoV-2 is distinct and holds a different position on the phylogenetic tree (Fig. 1). Whole-genome sequence alignments of SARS-CoV-2 with other CoVs have revealed that SARS-CoV-2 is most related to certain SARS-related-CoVs including bat CoVs (96%) and pangolin CoVs (86%-92%)^60^. For example, the whole genome sequence identity of SARS-CoV-2 with BatCoV RaTG13 is 96%, with SARS-CoV-1 79.6%, and with MERS-CoV 50%^61,62^.

The S protein comprises three segments: a large ectodomain, a single-pass transmembrane anchor, and a short intracellular tail. The ectodomain consists of a receptor-binding subunit S1 and a membrane-fusion subunit S2. The S is a clove-shaped trimer with three S1 heads and a trimeric S2 stalk^63–65^.

The D614G mutation was the first notable substitution in the S protein of SARS-CoV-2 and quickly spread worldwide^66^. Hereafter, multiple VOCs, including Alpha, Beta, Gamma, Delta, and Omicron, emerged independently across the globe^67^. One of the variants, Omicron (BA.1), which first appeared in Nov 2021 in Botswana, was antigenically distinct from previous VOCs and became dominant within a short span of time^68^. Following Omicron BA.1, several subvariants have appeared in different regions all around the globe^69–71^. Recombination events resulted in the appearance of additional VOCs^72^. During evolution, Omicron lineages exhibited antigenic shifts, resulting in poor neutralization by antibodies induced by first-generation vaccines and pre-Omicron infection^15,73^.

In the initial stages of the pandemic, the evolution of SARS-CoV-2 appeared to select for VOCs with higher transmissibility and, in some cases, higher severity^74–77^. Mutations resulting in antigenic changes leading to immune escape were recognized during the later stages of the pandemic^78^. Now, it is postulated that the evolution of SARS-CoV-2 is shifting towards adapting to different tissue tropisms as well as immune evasion^79^. For example, Omicron BA.1 has evolved a preference for efficient replication in the nasopharynx, a better vantage point for entering aerosols^80,81^. These changes may have a notable impact on the development of a successful vaccine that can prevent virus infection and transmission.

### Coronavirus vaccine development: the past and the present

HCoVs have been circulating in the population for decades, and no licensed CoV vaccine was available until December 2020 as a direct consequence of the COVID-19 pandemic. Before the pandemic, a few vaccine candidates against MERS-CoV, MVA-MERS-S, GLS-5300 DNA, and ChAdOx1-MERS were tested in phase I clinical trials^82–86^. Nevertheless, none were entered into phase II or III trials and approved for human use by the US FDA (United States Food and Drug Administration).

It has been 20 years since the scientific community has identified the potential of using S protein as a vaccine candidate against CoVs^87–89^. Multiple studies have demonstrated that S-specific antibodies can neutralize SARS-CoV-1 or MERS-CoV and protect animals^90–92^. Different variations on the S protein, including full-length, S1 domain only, or RBD, can be used to develop effective vaccines against SARS-CoV-2^20,93^. Moreover, a multitude of vaccines and vaccine delivery platforms have been developed, including mRNA, DNA, protein subunits, whole virus in inactivated form, live-attenuated virus, viral vectors, and lipid nanoparticles^94–98^. Most of the first-generation vaccines against SARS-CoV-2 are based on the ancestral Wuhan virus strain and use a prefusion stabilized S. Two mutations in the S2 subunit between the central helix (CH) and heptad repeat 1 (HR1), K986P and V987P, stabilize the prefusion confirmation and were critical for the effectiveness of COVID-19 vaccines^99^. Substitution of these residues was initially described for SARS-CoV-1 and MERS-CoV^100,101^. Subsequent studies showed that the S could be further stabilized by substituting six prolines, resulting in improved neutralizing antibody responses and efficacy^102,103^.

Eleven vaccines are approved for human use (Table 1) and more than 5 billion people are vaccinated with at least one dose of vaccine^104^. During the initial COVID-19 waves, multiple vaccines were shown to provide partial protection from infection, severe disease, and death^105–107^. mRNA-1273 and BNT162b2 vaccines, both mRNA vaccines, showed more than 90% efficacy in preventing symptomatic infection and disease severity^108,109^. AZD1222, a replication-incompetent adenovirus vaccine, prevented disease severity to 100%^110^. The efficacy of preventing symptomatic infection by a single dose of Ad26.CO.2 was 52.4%, and disease severity was 74.6%^108^. The mean efficiency of inactivated SARS-CoV-2 vaccines, including Covaxin, Covilo and CoronaVac, in preventing severe disease was 61.80%, 73.78% and 70.96%, respectively^111^. The emergence of VOCs completely altered this picture. Omicron VOCs have at least 32 mutations in the S protein and can partially escape the neutralizing antibody response elicited by Wuhan strain-based vaccines^15^. For instance, omicron subvariant BA.2.75 appeared early 2022, a descendent from BA.2, had several distinct mutations in its S protein, including five substitutions in the N-terminal domain (NTD), K147E, W152R, F157L, I210V, and G257S, and four substitutions in the RBD, D339H, G446S, N460K, and R493Q. BA.2.75 exhibited higher resistance to vaccine- and infection-induced serum neutralizing activity than BA.2, and the resistance was largely attributed to the K147E and N460K mutations^112^. Similarly, major genetic drift within the Omicron genome has resulted in the emergence of a highly mutated variant BA.2.86 in 2023 and replaced the circulating XBB and EG.5.1 variants. L455S, F456L, R346T, and D339H mutations within the S of BA.2.86 caused the emergence of a newer variant JN1 with reduced ACE2 binding and increased immune evasion^113–115^. These newer variants exhibited reduced neutralizing capacity within the convalescent and vaccinated individuals^116,117^.

**Table 1:** SARS-CoV-2 injectable vaccines granted emergency use listing by WHO

No.	Vaccine name	Manufacturer/Developer	Vaccine type
1	AZD1222/ Vaxzevria	AstraZeneca/Oxford Jenner Institute, UK	Nonreplicating viral vector (ChAdOx1)
2	AD5-nCoV/ Convidecia	CanSino Biological/Beijing Institute of Biotechnology/Academy of Military Medical Sciences, China	Nonreplicating viral vector (Ad5)
3	Covishield	AstraZeneca/ Oxford Jenner Institute, UK	Nonreplicating viral vector (ChAdOx1)
4	Ad26.COV2.S	Johnson & Johnson/Janssen Pharmaceuticals, USA/Belgium	Non-replicating viral vector (Ad26)
5	mRNA-1273/ Spikevax	Moderna/NIAID, USA	mRNA
6	BNT162b2/ Comirnaty	BioNTech/Pfizer, Germany/USA	mRNA
7	Covaxin/BBV152	Bharat Biotech/Indian Council Medical Research/National Institute of Virology, India	Inactivated SARS-CoV-2
8	BBIBP-CorV/ Covilo	Sinopharm CNBG/Beijing Institute of Biological Products, China	Inactivated SARS-CoV-2
9	CoronaVac	Sinovac Biotech, China	Inactivated SARS-CoV-2
10	NVX-CoV2372/ Nuvaxovid	Novavax	Inactivated SARS-CoV-2 protein subunit
11	COVOVAX	Serum Institute of India	Inactivated SARS-CoV-2 protein subunit

Breakthrough infections within vaccinated individuals by the Omicron variants substantiated that first-generation COVID-19 vaccines are inadequate in preventing transmission and the requirement of alternate strategies^118^. Despite the increase in antibody levels after breakthrough infection, neutralization titers against Omicron variants are significantly lower than the earlier variants^119–123^. To counteract the immunological imprinting effect of SARS-CoV-2, repeated Omicron boosting is required^124^. All these facts highlight the pressing need to develop next-generation vaccines that can broadly protect against novel variants and multiple related viruses from the same family.

It is important to emphasize that even though the neutralizing antibody response was reduced, all vaccine candidates continued to protect against severe disease caused by most of these VOCs. Protection could be via non-neutralizing antibodies^125–127^ or the induction of CD4+ and CD8 + T cells, for which epitopes are unaffected by S mutations^128–132^. S-specific CD4 + T cell memory responses induced by natural infection or mRNA vaccination are conserved against different VOCs, including Alpha, Beta, Gamma, and Omicron^133^. Despite partial escape of humoral immunity induced by SARS-CoV-2 infection or BNT162b2 vaccination by Alpha and Beta VOCs, S-specific CD4 + T-cell activation is not significantly affected by the mutations in these variants^134^. A similar study showed that SARS-CoV-2-specific memory CD4+ and CD8 + T cell recognition is not disrupted by the VOCs, including Alpha, Beta, and Gamma in COVID-19 convalescents and in recipients of the mRNA-1273 or BNT162b2 COVID-19 vaccines^135^. A study conducted in the BALB/c mouse model showed that T cell-based SARS-CoV-2 spike protein vaccine could provide significant protection against SARS-CoV-2 and Omicron BA.5 variant infection without inducing any specific antibodies. Moreover, the depletion of CD4+ or CD8 + T cells led to a significant loss of protection, indicating the role of T cells in limiting infection^136^. Patients with X-linked agammaglobulinemia without B cells can develop pneumonia and still recover from SARS-CoV-2 infection^137^. Computational tools have been developed to predict immunogenic and conserved T-cell epitopes, thus augmenting the precise selection of SARS-CoV-2 vaccine targets^138^. Thus, incorporating more T-cell epitopes while developing a vaccine would be able to provide more breadth and durability.

### The future of CoV vaccines

Organizations like the Coalition for Epidemic Preparedness Innovations (CEPI) and the National Institute of Allergy and Infectious Diseases (NIAID) have taken the initiative to provide funding for the development of advanced vaccines. These vaccines aim to offer protection against all current and future variants of CoVs by stimulating durable, broad-spectrum immunity. Researchers are updating available vaccines or considering an entirely new approach to achieve this goal.

Since developing a vaccine that protects against all CoVs is likely difficult to achieve due to its genetic variability, as discussed above, a tiered approach likely has a higher chance of success, beginning with COVID-19 vaccines, which can protect against all VOCs, to pan-sarbecovirus vaccines, to pan-betacoronavirus vaccines, to universal or pan-coronavirus vaccines^139^. Both Pfizer and Moderna have developed bivalent vaccines targeting ancestral SARS-CoV-2 and Omicron. Compared to the monovalent booster, these second-generation vaccines have moderately higher or similar effectiveness in inducing neutralizing antibody responses against Omicron variants in vaccinated infection-naïve cohorts^140–142^. However, immune imprinting and the antigenic variation within Omicron limit the induction of neutralizing antibody responses against newer variants^143–147^. A recent study carried out on serum samples obtained from participants who had received one or two monovalent boosters or the mRNA bivalent booster indicated that the neutralizing antibody responses are lower against BA.1, BA.5, BA.2.75.2, BQ.1.1, and XBB as that of the ancestral strain WA1 in all three cohorts^148^.

Other groups have focused on the development of broadly neutralizing sarbecoviruses vaccines by including multiple different antigens. Mosaic-8b, a multivalent spike receptor-binding domain nanoparticle vaccine displaying RBDs from SARS-CoV-2 and seven animal sarbecoviruses, was found to induce broadly neutralizing antibody responses and protected K18-hACE2 mice and non-human primates from SARS-CoV-1 and SARS-CoV-2 challenge, even though SARS-CoV-1 was not included in the vaccine. In contrast, nanoparticles with SARS-CoV-2 RBDs protected only against SARS-CoV-2 challenge. Compared to homotypic RBD nanoparticles expressing only SARS-CoV-2, immunization with mosaic-8b resulted in more robust binding and neutralizing antibody responses against SARS-CoV-1, SARS-CoV-2, and SARS-CoV-2 VOCs^149^. Another multivalent spike receptor-binding domain nanoparticle vaccine, GBP511, contains RBDs from SARS-CoV-2, SARS-CoV-1, and the bat CoVs WIV1 and RaTG13. This vaccine reduced SARS-CoV-1-MA15 virus replication in lung tissue of vaccinated mice. Furthermore, it elicited broad neutralizing antibody responses against multiple sarbecoviruses after a single immunization^150^. Likewise, a chimeric spike mRNA vaccine containing RBD, NTD, and S2 domains of various SARS-related CoVs could protect aged mice against SARS-CoV-1, SARS-CoV-2, SARS-CoV-2 Beta, bat CoVs RsSHC014, and a heterologous WIV-1 challenge^151^. Spike Ferritin Nanoparticle (SpFN) COVID-19 vaccine comprises S from multiple CoVs linked to the surface of a multifaceted ferritin nanoparticle and utilizes Army Liposome Formulation containing QS-21 (ALFQ) as an adjuvant. Upon vaccination, broadly neutralizing antibody responses against major SARS-CoV-2 VOCs and SARS-CoV-1 were induced, resulting in protection against ancestral SARS-CoV-2 in NHPs^152,153^. Finally, RBD-sortase-A-conjugated ferritin nanoparticle (RBD-scNP) vaccine protects both NHPs and mice from multiple CoV challenges^154,155^. RBD-scNP contains recombinant SARS-CoV-2 RBD with a C-terminal sortase A donor sequence and self-assembling Helicobacter pylori ferritin with an N-terminal sortase A acceptor sequence.

Antigen design may be important in inducing a broadly protective immune response. DIOSynVax is developing an mRNA vaccine, T2_17, based on the common viral structures on all sarbeco viruses reducing the need for synthesizing, processing, and manufacturing multiple components within one vaccine. A membrane-anchored form (T2_17_TM) mRNA immunogen was shown to be immunogenic against SARS-CoV-1, SARS-CoV-2, WIV16, and RaTG13 in BALB/c mice, guinea pigs, and outbred rabbits. Challenge studies on K18-hACE-2 mice primed with AZD1222 vaccine and boosted with either AZD1222 or T2_17 as an MVA-vectored immunogen showed induction of neutralizing antibody responses against pseudoviruses of SARS-CoV-1, SARS-CoV-2 and the Delta VOC^156^.

**Fig. 2:**
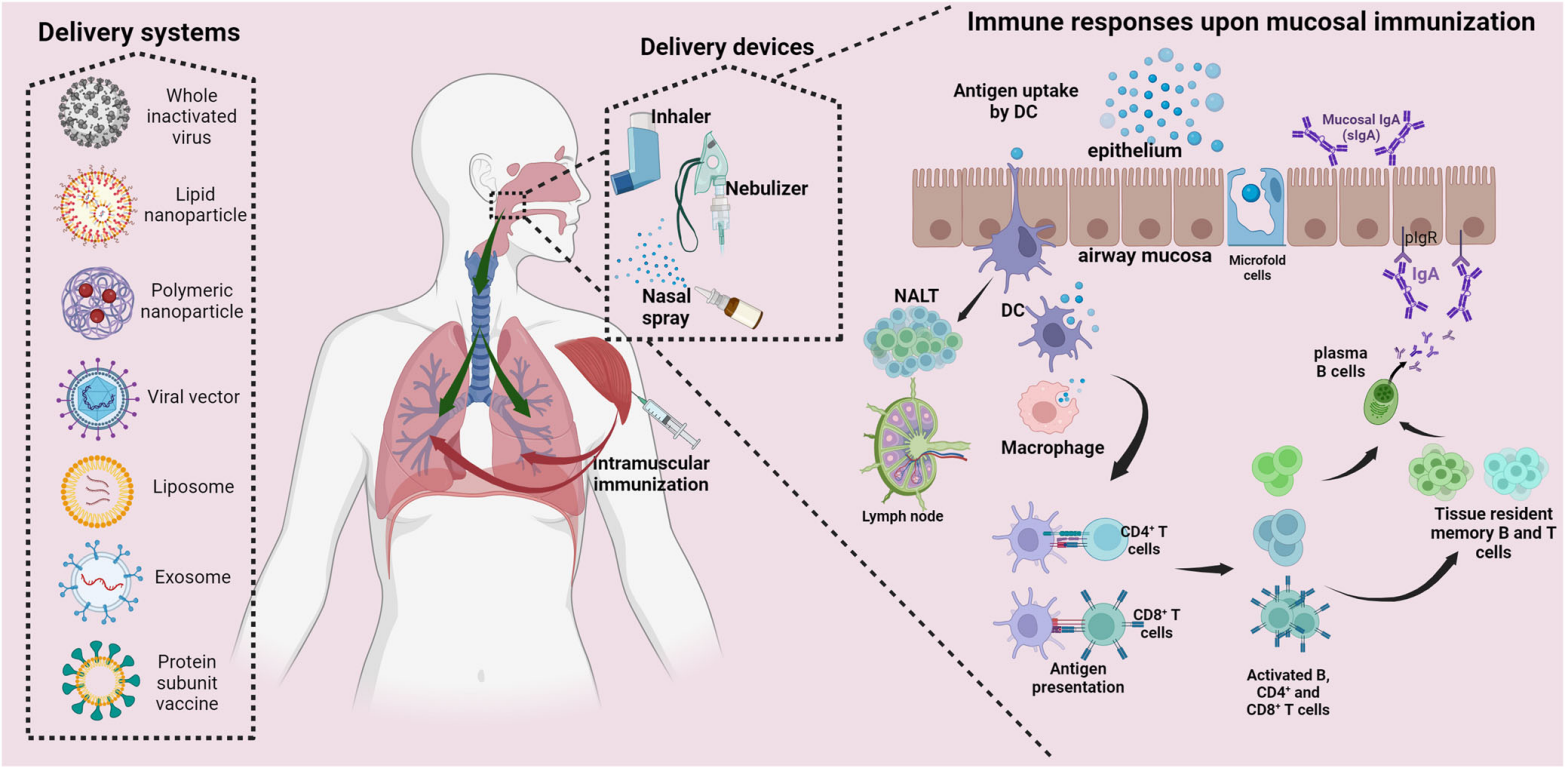
Different vaccination strategies and the resulting immune responses. Various delivery systems currently used to administer vaccines are shown on the far left. Vaccine delivery routes and devices are depicted in the middle, and the resulting immune pathway after intranasal inoculation is shown on the right. Intranasal delivery of vaccine is achieved by using an inhaler, nebulizer, or spray method. Vaccination via the mucosal route can mount an immune response both in the upper (URT) and lower (LRT) respiratory tract (indicated by green arrows). Microfold cells present on the nasal mucosa actively transport antigens to the dendritic cells (DCs) and macrophages in the subepithelial space. Additionally, DCs situated in the lamina propria or interspersed among epithelial cells sample the mucosal environment using extensions. Activated DCs and macrophages migrate to regional draining lymph nodes or tertiary lymphoid follicles and present antigens to T and B cells. Resulting effector T cells may traffic to the respiratory tract as tissue-resident memory T cells (TRM). Activated B cells either differentiate into low-affinity IgG or IgA-producing plasma cells, which traffic to the respiratory tract, or move to the germinal center, undergo class-switching and somatic hypermutation, and differentiate into long-lived plasma or memory B cells, secreting high-affinity immunoglobulins. These memory B cells may traffic to the respiratory tract. Here, IgA is mainly produced in its polymeric form (pIgA), predominantly dimeric, and transported across the epithelium of the respiratory tract by polymeric Immunoglobulin receptor (pIgR). The pIgR-pIgA complex is cleaved at the apical surface of the epithelium. Thereby, IgA gains part of the pIgR named the secretory component and is released as secretory IgA (sIgA). The secretory component increases the stability of sIgA. Intramuscular immunization can mount robust systemic and LRT immune responses (indicated by red arrows). Circulating IgG antibody levels are generally higher upon vaccination via the intramuscular route in comparison with the intranasal route. NALT Nasal-associated lymphoid tissue. Created with BioRender.com.

Several studies indicated that intranasally (IN) administered vaccines could induce comparable systemic humoral and cellular immunity as IM vaccination and protect the upper and lower respiratory tract of animals against SARS-CoV-2 infection (Fig. 2)^24,157–162^. Moreover, reduced nasal shedding of the virus was observed upon IN challenge, suggesting that mucosal vaccines could be better at reducing SARS-CoV-2 transmission^163,164^. Dimeric IgA in the nasopharynx has higher neutralizing capability than monomeric IgA and IgG^165^. A recent study conducted to evaluate the effectiveness of intranasal administration of sCPD9-ΔFCS, a replication-competent yet fully attenuated virus vaccine, showed induction of robust IgA in nasal washes, which neutralized Omicron BA.1 and BA.5, and limited virus transmission in hamsters models^165,166^.

More than 20 mucosal vaccine candidates are in development and clinical trials, and two are approved for human use. iNCOVACC (BBV154) is an intranasal SARS-CoV-2 vaccine developed by Bharath Biotech, which was approved in India in early 2023 as a booster dose for those above 18 years of age^167^. iNCOVACC is a recombinant replication-deficient adenovirus-vectored vaccine encoding a pre-fusion stabilized SARS-CoV-2 S formulated as nasal drops to allow IN delivery. A preclinical study on mice expressing the hACE2 receptor indicated that a single IN dose of iNCOVACC induced high levels of neutralizing antibodies in serum and anti-SARS-CoV-2 IgG and IgA levels in serum and BAL fluid. Challenge studies confirmed complete protection against SARS-CoV-2 infection in the upper and lower respiratory tracts^159^. Further comparative clinical trial data on healthy adults showed that salivary IgA levels were higher in those who received two doses of iNCOVACC IN, 28 days apart, compared to the licensed intramuscular vaccine, Covaxin. Both vaccines induced significant serum-neutralizing antibody responses against ancestral SARS-CoV-2 and Omicron BA.5^167^.

A recombinant adenovirus type-5 vectored COVID-19 vaccine (Ad5-nCoV) has been authorized for use as a prime and booster dose in China^168^. This vaccine is administered via oral inhalation using a nebulizer to deliver the vaccine as aerosol particles into the lungs. Ad5-nCoV is highly immunogenic in clinical trials and efficacious in preventing severe COVID-19^169–172^.

IN administration of AZD1222, currently approved as an IM vaccine, can reduce viral shedding and protect hamsters and non-human primates from SARS-CoV-2 challenge^163^. A phase I clinical trial to investigate the mucosal and systemic immune responses elicited by intranasal AZD1222 as a booster was not encouraging, possibly due to the low number of participants and lack of proper control groups, and more studies have to be conducted to draw final conclusions^173^.

Two additional nasal vaccine candidates, NDV-HXP-S and CoviLiv, have completed phase I clinical trials^174^. NDV-HXP-S, a recombinant Newcastle disease virus-based vaccine, which expresses HexaPro S, has shown enhanced neutralizing and binding antibodies from serum samples obtained postvaccination in comparison with samples from mRNA BNT162b2 vaccinees^175^. A live-attenuated COVID-19 candidate, CoviLiv by Codagenix, expresses all SARS-CoV-2 proteins. IN administration of this vaccine in unvaccinated/uninfected volunteers induced mucosal immunity in a phase I clinical trial. Moreover, participants who received two doses of CoviLiv showed induction of both humoral and cellular immune responses^176^.

Most mucosal vaccine data comes from preclinical studies. IN vaccination with an N1-methyl-pseudouridine–modified mRNA-LNP results in decreased viral loads in the respiratory tract and reduced lung pathology in Syrian hamsters after the SARS-CoV-2 challenge compared to IM controls^177^. Vaccination of IFNAR1 − /− mice and hamsters using IN trivalent measles-mumps-SARS-CoV-2 spike protein vaccine induced robust neutralizing antibody responses, mucosal IgA, and systemic and lung resident T cell immune responses and resulted in protection against three different variants of SARS-CoV-2^178^. An alternate vaccine-boosting strategy, a primary IM vaccination with an mRNA vaccine (“prime”), and an intranasal dose of unadjuvanted SARS-CoV-2 S (“spike”) showed robust cellular and humoral mucosal immune responses in K18-hACE2 mice and protected the animals against SARS-CoV-2 infection. The same strategy reduced viral shedding and prevented transmission in the Syrian hamster model compared to naïve animals^24^. Finally, MigVax-101 is an orally administered disintegrating freeze-dried tablet vaccine that aims to provide mucosal and humoral immune responses and offers advantages, including ease of administration, low cost, and easy storage. MigVax-101 is a multi-antigen vaccine that contains the RBD and two domains of the N from SARS-CoV-2 and heat-labile enterotoxin B (LTB) as a potent mucosal adjuvant. Immunization studies performed in BALB/c mice and Sprague Dawley rats using MigVax-101 as a homologous oral vaccination of three doses and a heterologous subcutaneous prime and oral booster regimen indicated potent humoral, mucosal, and cell-mediated immune responses compared with the control animals^179^.

### Future challenges

The continuous evolution of SARS-CoV-2 has posed several challenges and raised serious concerns about the effectiveness of currently available vaccines regarding virus infection and transmission. We posit that the development of broad vaccines with a mucosal immune activation component would be ideal for coronaviruses, along with other respiratory viruses.

Several challenges need to be addressed for both the development and clinical testing of vaccines. For example, the human population now exhibits extensive heterologous immunity, and it is not well understood how this affects subsequent vaccination. Furthermore, efficacy testing of novel vaccines in Phase III clinical trials is hindered by this existing immunity, as there will be no placebo group to compare infection rates to. Identifying correlates of protection is necessary to determine a vaccine’s effectiveness against SARS-CoV-2. Another intriguing question is how one can test the efficacy of a vaccine against an as-of-yet-unknown SARS-CoV-3, and likely fully hinges on understanding the correlates of protection.

As detailed above, mucosal vaccination is a promising and logical approach to induce immunity in the respiratory tract. However, more research is required to understand the specific correlates of protection in the respiratory tract, as these are currently unknown. Furthermore, due to previous issues with adjuvants, extra attention should be paid to the safety of mucosal vaccine candidates^180^. Factors to consider on the way to a successful mucosal vaccine include safe antigens and adjuvants, which vaccine platforms are best at inducing mucosal correlates of protection, what animal models can be used to test efficacy and safety, and what delivery devices are optimal. Another hurdle in developing a nasal vaccine is to address nasal clearing of the vaccine by mucus and cilia. Both mucus and cilia act as a protective barrier in the nasal mucosa and adversely affect antigen absorption. One way to address this issue would be by using virus-like particles and replication incompetent viral vectors that can infect the nasal epithelium or by using substances like gel that can remain in the nasal mucosa for a longer period to ensure proper antigen absorption.

In this review, we have highlighted the pressing need for pan-CoV vaccines against existing and novel emerging CoVs. Developing new tools and finding new vaccination strategies will be an effective way to achieve broad protection against existing and emerging coronaviruses. Multiple broad-spectrum vaccines are currently in various stages of development. Choosing alternative routes for vaccine delivery, along with proper immunogen design, are key approaches to move toward the development of broad-spectrum vaccines. Generating a vaccine against conserved epitopes within CoVs could provide a broader range of protection. The comparative effectiveness of IM and IN vaccination routes is debatable; however, designing and administering vaccines using both routes is an efficient way forward. Combining both routes can theoretically protect individuals from disease severity and virus transmission. Since the population has already received at least one dose of the IM vaccine, a potential IN booster in the form of a self-administrable nasal spray or inhaler would be an intriguing option. More must be addressed before moving into the next step of vaccination in humans.

The COVID-19 pandemic showcased the rigor and potential of the scientific community in developing, testing, and deploying multiple vaccine candidates within an unprecedented timeframe, achieving milestones in the history of vaccines. The COVID-19 pandemic also reminds us of the importance of global pandemic preparedness and the need to invest in research and development. Although COVID-19 is no longer considered a serious threat, the development of innovative vaccine formulations and delivery strategies resulting in improved immunogenicity, safety, and efficacy could make a significant impact on future pandemics.
